# Supplementary material for: Greener Synthesis of Pristane by Flow Dehydrative Hydrogenation of Allylic Alcohol Using a Packed-Bed Reactor Charged by Pd/C as a Single Catalyst
Source: Molecules. 2021 Sep 27;26(19):5845. doi: 10.3390/molecules26195845 (PMC8510359; doi:10.3390/molecules26195845)
Supplement: Supplementary file 1 [file molecules-26-05845-s001.zip › molecules-1343388-supplementary.pdf]

**Supporting Information**

# Greener Synthesis of Pristane by Flow-Dehydration-Hydrogenation of Allylic Alcohol Using a Packed-Bed Reactor Charged by Pd/C as a Single Catalyst

**Takayoshi Kasakado,[1] Yuki Hirobe,[2] Akihiro Furuta,[2] Mamoru Hyodo,[1] Takahide Fukuyama,[2,*] and Ilhyong Ryu[1,3*]**

[1] Organization for Research Promotion, Osaka Prefecture University, Sakai, Osaka 599-8531, Japan
[2] Department of Chemistry, Osaka Prefecture University, Sakai, Osaka 599-8531, Japan
[3] Department of Applied Chemistry, National Yang Ming Chiao Tung University (NYCU), Hsinchu 30010, Taiwan

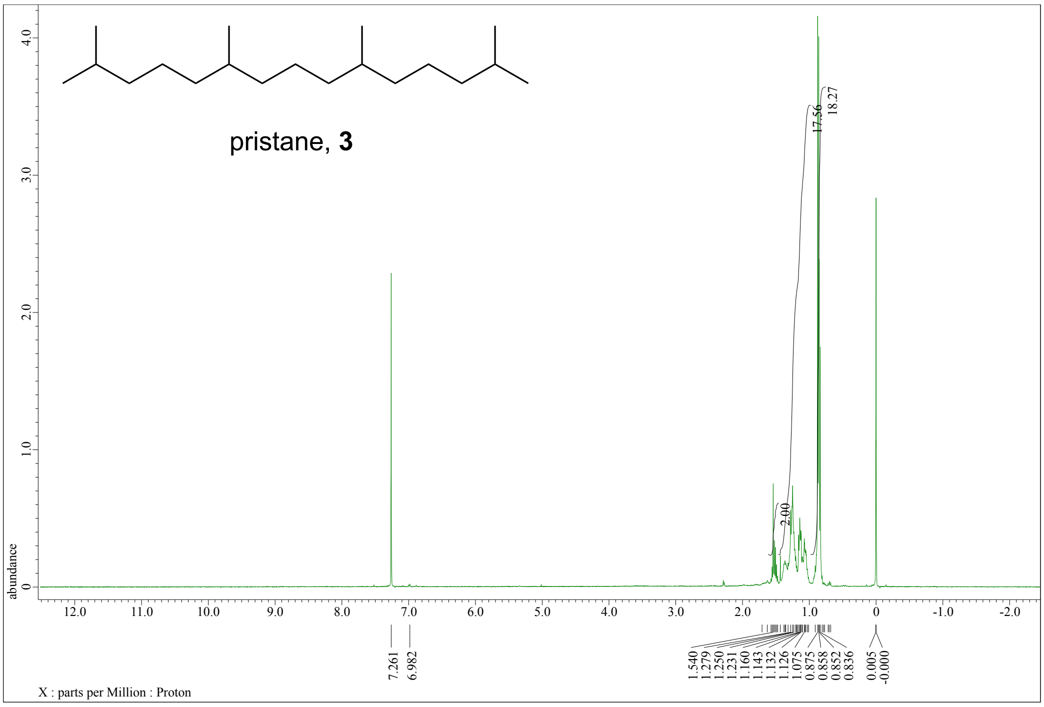
pristane, 3
abundance
17.56
18.27
2.00
7.261
6.982
1.540
1.279
1.250
1.231
1.160
1.143
1.132
1.126
1.075
0.875
0.858
0.852
0.836
0.005
-0.000
X : parts per Million : Proton

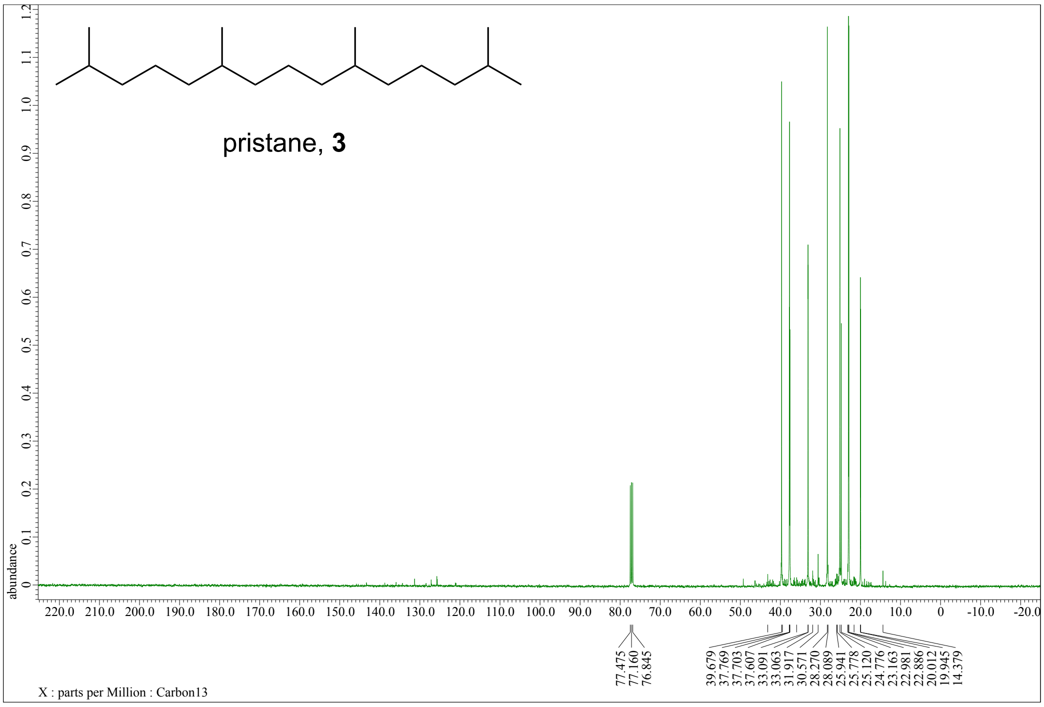
pristane, 3
abundance
77.475
77.160
76.845
39.679
37.769
37.703
37.607
33.091
33.063
31.917
30.571
28.270
28.089
25.941
25.778
25.120
24.776
23.163
22.981
22.886
20.012
19.945
14.379
X : parts per Million : Carbon13

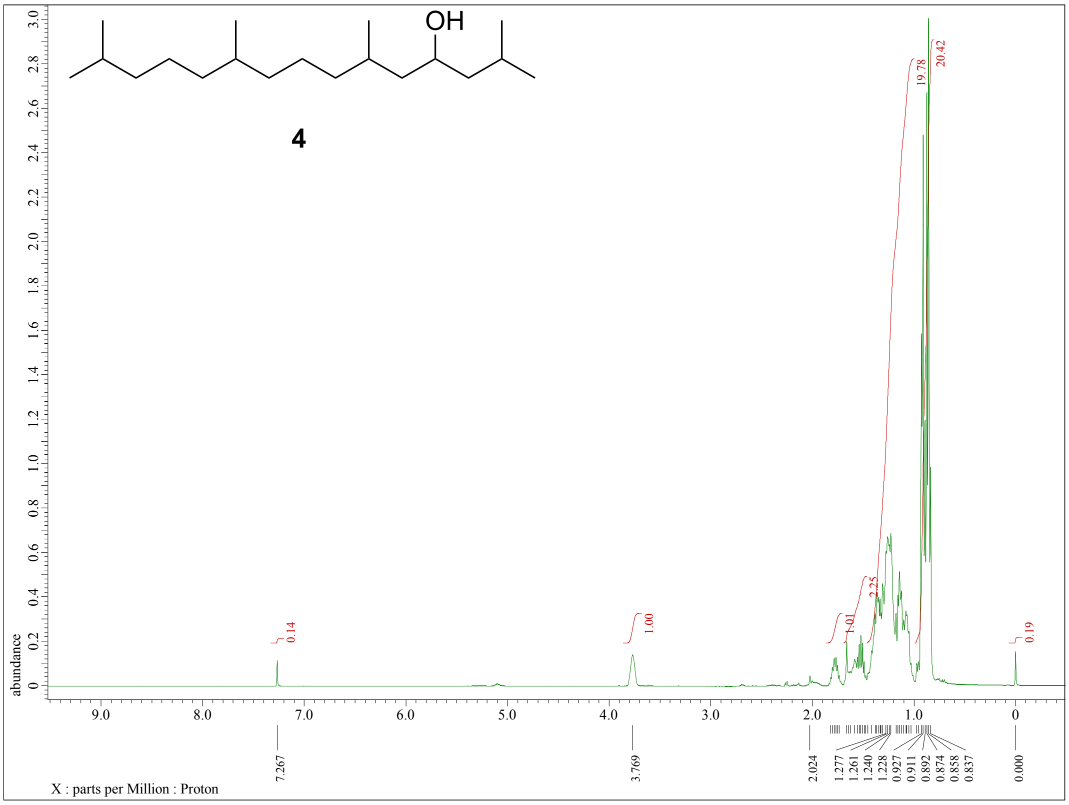
OH
4
abundance
X : parts per Million : Proton
0.14
1.00
1.01
2.25
19.78
20.42
7.267
3.769
2.024
1.277
1.261
1.240
1.228
0.927
0.911
0.892
0.874
0.858
0.837
0.000

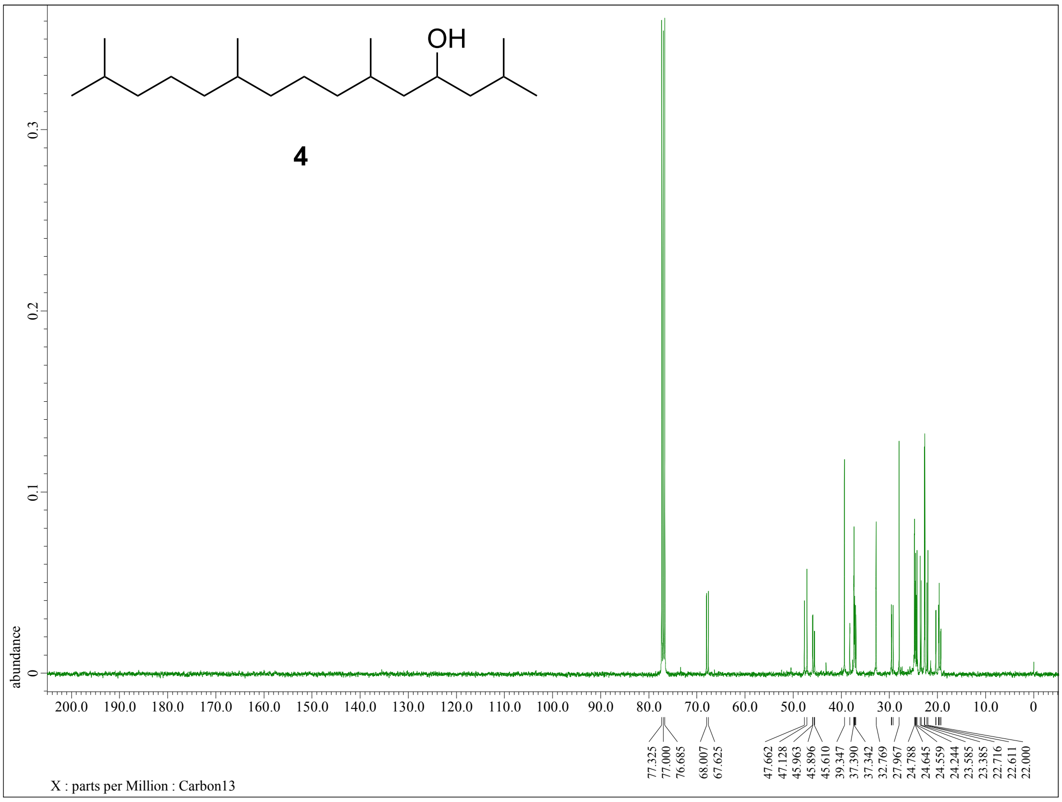
OH
4
abundance
X : parts per Million : Carbon13
77.325
77.000
76.685
68.007
67.625
47.662
47.128
45.963
45.896
45.610
39.347
37.390
37.342
32.769
27.967
24.788
24.645
24.559
24.244
23.585
23.385
22.716
22.611
22.000
